# Supplementary figures and images for: Psychopathy, pain, and pain empathy: A psychophysiological study
Source: PLoS One. 2024 Jul 5;19(7):e0306461. doi: 10.1371/journal.pone.0306461 (PMC11226074; doi:10.1371/journal.pone.0306461)

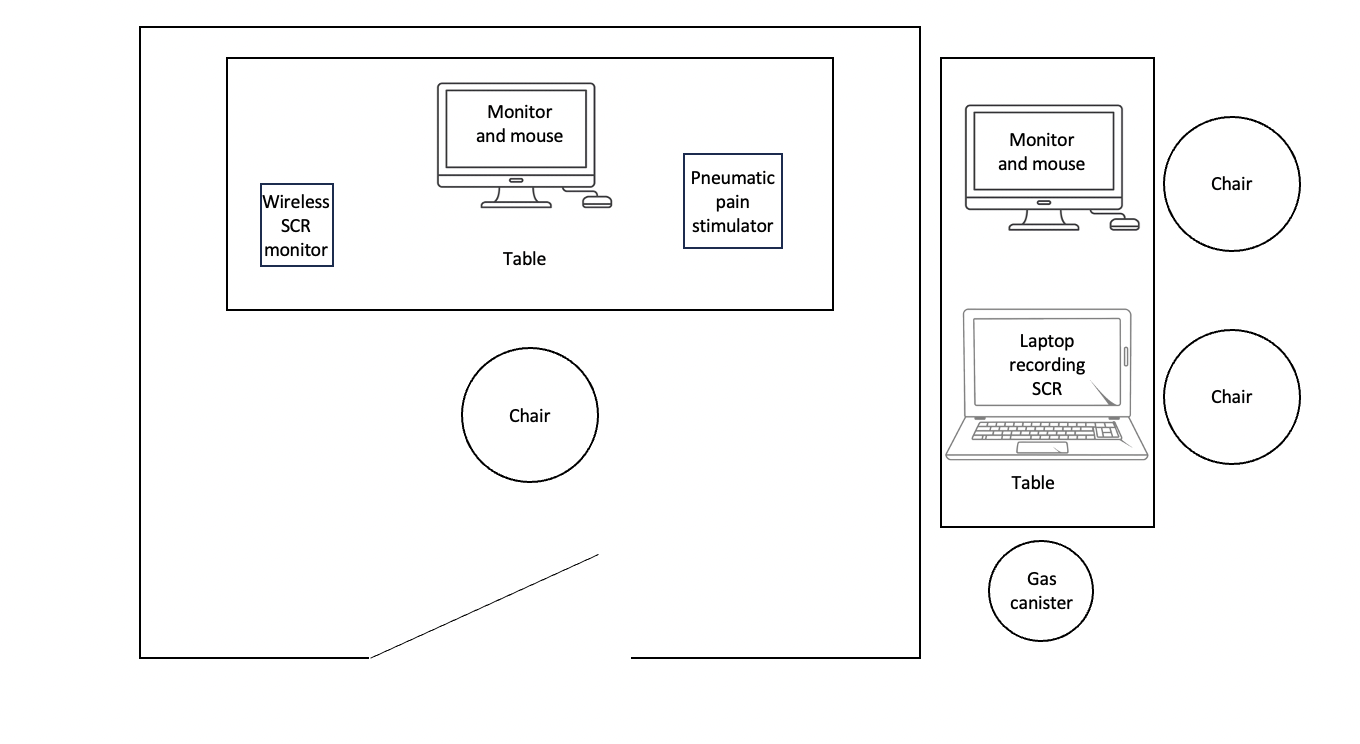


**S1 Fig.** Diagram representing the layout of the experimental setup in the laboratory.

Supplement: S1 Fig — (DOCX) [file pone.0306461.s001.docx]
