# Supplementary material for: Psychopathy, pain, and pain empathy: A psychophysiological study
Source: PLoS One. 2024 Jul 5;19(7):e0306461. doi: 10.1371/journal.pone.0306461 (PMC11226074; doi:10.1371/journal.pone.0306461)
Supplement: S3 Fig — a–presentation of image; b– 1–4 second time window when any increase (over 0.1 microsiemens) in SC was (d) of an ER SCR; c–amplitude of ER SCR. (DOCX) [file pone.0306461.s003.docx]

**
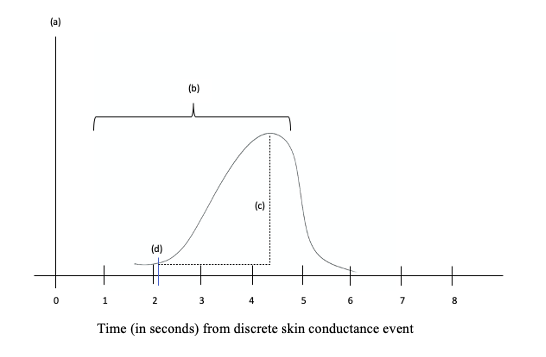
**

**S3 Fig**. **Timeline for event-related (ER) analysis for empathy task**: *a* – presentation of image; *b* – 1-4 second time window when any increase (over 0.1 microsiemens) in SC was (*d*) of an ER SCR; *c* – amplitude of ER SCR.
