# Supplementary material for: Psychopathy, pain, and pain empathy: A psychophysiological study
Source: PLoS One. 2024 Jul 5;19(7):e0306461. doi: 10.1371/journal.pone.0306461 (PMC11226074; doi:10.1371/journal.pone.0306461)
Supplement: Attachment — Submitted filename: Review_attachment.pdf [file pone.0306461.s005.pdf]

The present manuscript investigates the relationship between psychopathy, pain and empathy using both self-reports and psychophysiological measures. The results indicate less self-reported physical pain and empathy in people higher in psychopathy, compared to those lower in psychopathy. Notably, the results obtained using skin conductance response (SCR) to physical pain did not point to differences between people residing in highest and lowest fifth of the psychopathy spectrum.

These main results of the study are highly significant for the discipline as they suggest distinct evaluation of pain in people with psychopathic traits, with only subjective experiences of pain distinguishing people higher and lower in psychopathy. Regarding empathy, both objective and subjective measures suggested distinct responses in people residing in highest and lowest fifth of the psychopathy spectrum.

Both the identified research gaps (see Introduction) and the findings of the study (see Discussion) are properly placed in the context of the previous literature, considering a wide range of studies conducted in the field. Furthermore, the data and analyses fully support the authors' conclusions. The procedure along with the utilised measures and analyses is described in sufficient detail, ensuring transparency and comprehensibility of the methods used. Finally, the authors discuss the implications of these findings in an intelligible fashion and propose several ideas for future research.

Taken together, the manuscript is well organized, clearly written and scientifically sound. However, there are some points that should be addressed prior to accepting the paper for publication:

Abstract:

- Consider replacing "psychophysiology" with "psychophysiological measures" in the first paragraph.
- Consider replacing "We suggest psychopathy interventions focus on recognising and empathising with pain." with "Psychopathy interventions should thus focus both on recognising and empathising with pain."

Introduction:

- Please add a reference after the first sentence.
- Page 3: Instead of "Why look into pain?", use the following wording: "There are several reasons for investigating pain in the context of psychopathy." to ensure a smooth transition between the first and second paragraph.
- Page 4: Since *Plos One* is a multidisciplinary journal, I think it would be helpful to shortly explain the meaning of "manual and automated pressure algometers" and "Skin Conductance Response".
- Page 4: The part "reduce the likeliness of socially desirable responding" is somewhat confusing and misleading as socially desirable responding represents a bigger problem in self-report measures compared to psychophysiological measures. It should be clear that the part "reduce the likeliness of socially desirable responding" refers to the comparison between mail, web and telephone approaches (see reference Nr. 14), and not to the comparison between self-report and psychophysiological measures.

- Page 5: In the sentence “The present study sought to…” please use “self-reported measures” or “self-reports” instead of just “self-reported”.
- Page 5: In my opinion, the two questions (H_1 and H_2) represent the research questions and not the hypotheses. Thus, I would suggest to rephrase this part as follows: “Following research questions were addressed:
  1) Do people higher in psychopathy experience less intense pain than people lower in psychopathy?;
  2) Do people higher in psychopathy feel less empathy for other people’s pain?
  Alternatively, please rephrase the questions to make them sound more like hypotheses, e.g. If people are higher in psychopathology, then they will experience less intense pain than people lower in psychopathy.

Methods:

- Page 5: Add “on” before “notice boards”.
- Page 5: The opening parenthesis is missing before YPI.
- Page 5: You write that 131 participants were contacted to take part in the laboratory experiment. It would be good to also note how many of them were in the highest and how many in the lowest 20% of the psychopathy spectrum.
- Page 5: I suggest moving the part “aged between…” right after the parenthesis with the gender distribution, namely: “a total of 49 adults (female *n* = 26; male *n* = 23), aged between 15-55 years old…”.
- Though both forms are used in standard English, I would suggest using “data were” rather than “data was”, since the former is more common in scientific articles.
- Page 5: Please specify which data (e.g. sociodemographic data) were anonymised. On the next page, you write that <u>the data from the tasks</u> were anonymised, but here it remains unclear which data were meant.
- Page 6: It would be helpful to add a figure or a picture to illustrate the setting of the experiment. The written explanation is very well written, but a graphical representation would be a nice addition, especially considering the multidisciplinary nature and the wide audience of the journal.
- Page 6: Please specify which two self-report questionnaires were given to the participants to accommodate a 10-minute stabilisation period for SCR.
- Page 6: To enhance readability, please add “stimulations” after “10 touch pain” and “10 pain threshold”.
- Page 7: If possible, please indicate *M* and *SD* for the duration of the tasks.
- Page 7: Please provide a brief explanation for “phalanges” to make the word understandable for a wide audience.
- Page 9: Add a space between 1 and cm^2 (in “1cm^2 circular probe”).
- Page 10: Please add the Cronbach’s alpha values for the YPI and its subscales. This has been done for other questionnaires, but not for the YPI.

- Page 11: When describing the Likert scale used in the IRI, double quotation marks were used, i.e. “does not describe me well” (0) to “describes me very well” (4). In previous questionnaire descriptions, you used single quotation marks. Please revise this for consistency.
- Page 12: In “…and SCR and self-report responses” do you refer to the empathy measure? Or did I misunderstand something?

Results:

- Italics are not consistently used for letters used as statistical symbol (e.g. page 13, t (47) = 0.51; page 15, paragraph “We also tested if the pain images…”). Please go through the text and make sure italics are consistently used for statistical symbols.
- Page 12: The formulation “to bring the skewness to a more normal level” is rather suboptimal. Please consider another wording, e.g. “to ensure that the data follow approximately normal distribution”.
- Page 13: To better align the wording of the question and the first sentence, consider replacing “greater” with “lower” in the first sentence.
- Page 14: For consistency, please replace “SC response” with “SCR”.
- Page 14: In the sentence “High pain intensity was rated…” please add “level” after “at significantly higher”, for the sake of clarity.

Discussion:

- Page 15: In the first sentence “Our study aimed to…” please consider alternative wording for “psychopathy”, e.g. psychopathic traits, psychopathy level.
- Page 15-16: The sentence “Lastly, we also show…” seems misplaced. In my opinion, it is rather confusing to mention criminal and non-criminal psychopaths this early in the discussion. I suggest deleting the sentence, since it is sufficient to elaborate on this later in the discussion.
- Page 16: In the sentence “Yet, people higher in…” add “is” after “pain threshold which”.
- Page 16: There is no need to abbreviate callous-unemotional and blood pressure. There are already many abbreviations used on this page and additional abbreviations only interrupt the reading flow.
- Page 16: The word “however” appears three times in the last six lines. Please consider rephrasing the sentences or replacing “however” with a synonym (e.g. nevertheless) on some occasions.
- Page 18: The internal consistency of some measures was not satisfactory. Please address this in the limitations.
- Page 19: Add a comma after “a finger trapped in a door”.
- Page 19: Replace “helps to add to literature” with “adds to literature”.
- Page 19: Instead of “This is important as interventions…” please write “This is of high clinical/practical relevance since interventions…”. This way, you avoid repeating the phrase “it is important” and improve the reading flow.

- Page 19: Consider replacing the sentence “This is important when developing…” with “When developing interventions targeting empathy deficits, a focus should lie on recognising and empathising with pain.”

Overall, this is an interesting and well-written study. I hope this feedback will help the authors further improve the quality of the manuscript which, in my opinion, could be accepted after a minor revision
