# Supplementary material for: Psychopathy, pain, and pain empathy: A psychophysiological study
Source: PLoS One. 2024 Jul 5;19(7):e0306461. doi: 10.1371/journal.pone.0306461 (PMC11226074; doi:10.1371/journal.pone.0306461)
Supplement: Attachment — Submitted filename: Response to reviewers FINAL.docx [file pone.0306461.s006.docx]

Dear reviewers,

Thank you very much for the comments you have made on our manuscript. We appreciate the feedback and have hopefully addressed all the comments that have been made to the correct standard. Below, you will find each of the comments that we received and the action we have taken.

Thank you for your time and effort when reviewing our work.

5. Review Comments to the Author

Please use the space provided to explain your answers to the questions above. You may also include additional comments for the author, including concerns about dual publication, research ethics, or publication ethics. (Please upload your review as an attachment if it exceeds 20,000 characters)

**Reviewer #1:** The topic of the manuscript concerns the relationship between own pain, pain in others and psychopathic traits. There are strengths to the manuscript, but also major issues that need to be addressed. I have outlined them below.

INTRODUCTION
1) The writing is good in general, but the organisation of the introduction can be improved. I do not intend to sound harsh, but I was sometimes left with the impression that this was initially a thesis that was turned into an article. Adding more depth to the introduction and also logical transitions between paragraphs would help improve the introduction.

Response: Thank you for your comment. We have added more depth and logical transitions to the introduction.

2) First sentence: Psychopathy is not a single personality trait, but a constellation of multiple traits. In general, the operationalization of the construct needs to be more elaborate, especially given that many of the studies cited in the manuscript use other operationalizations and measurements of psychopathy. Also, the idea that such traits can be measured in both criminal and non-criminal population needs to be explained clearly.

Response: Thank you for your comment. We have expanded our reading and added more research to help support our argument.

3) The manuscript would benefit greatly from a much more thorough coverage of research on pain empathy and psychopathy. Several key references are lacking and not discussed. For example, the study by Brazil, Atanassova & Oosterman (2022) addressed this same topic and is not even mentioned, even though it is perhaps the only published study to date examining links between psychopathy, own pain experience and experience of pain in others. The work by Bird and Viding (2014) is also highly relevant, as it provides a clear theory on how own emotions are the basis of empathy, which was explicitly linked to psychopathy (now the authors extrapolate claims from alexithymia research to say this psychopathy). This would help make the conceptual embedding offered in the introduction (and the discussion) much richer, thorough and balanced.

Response: Thank you for your comment. Brazil et al (2022) and Bird & Viding (2014) have been read and included in the introduction to help the conceptual embedding.

4) p3: '..it would be interesting to see how an individual’s psychopathy level relates to directly experienced pain...'. Whether something is interesting or not is subjective and not a solid ground for research. This makes the rationale for conducting the study less compelling. The authors should provide an argument based on empirical grounds instead. This requires a firmer embedding in prior research to identify the gaps of knowledge.

Response: Wording of the last sentence changed.

5) There are multiple claims made that are innacurate. For example, '...it has rarely been used to investigate the links between pain and psychopathy.' Such statements are not entirely accurate. There are definitely studies using psychophysiology (and even MRI) to assess own pain and/or pain in social context in relation to psychopathy. The authors should conduct a much more thorough search for articles and find a way to incorporate the papers they find into the manuscript to add nuance and depth and use them to articulate their reasoning more clearly. There is no need to be overly selective in discussing and citing studies. Some examples for where to start looking are:

Response: Statement containing “rarely been used” amended. Papers recommended have been read to increase breadth and depth of knowledge, and some have been included in the manuscript.

Hare, R. D. (1965). Psychopathy, fear arousal and anticipated pain. Psychological Reports, 16, 499–502.

Marcoux, L.-A., Michon, P.-E., Voisin, J. I., Lemelin, S., Vachon-Presseau, E., & Jackson, P. L. (2013). The modulation of somatosensory resonance by psychopathic traits and empathy. Frontiers in Human Neuroscience, 7. https://doi.org/10.3389/fnhum.2013.00274

Brazil, I. A., Atanassova, D. V., & Oosterman, J. M. (2022). Own Pain Distress Mediates the Link Between the Lifestyle Facet of Psychopathy and Estimates of Pain Distress in Others. Frontiers in Behavioral Neuroscience, 16. https://www.frontiersin.org/article/10.3389/fnbeh.2022.824697

Decety, J., Skelly, L. R., & Kiehl, K. A. (2013). Brain Response to Empathy-Eliciting Scenarios Involving Pain in Incarcerated Individuals With Psychopathy. JAMA Psychiatry, 70(6), 638–645. https://doi.org/10.1001/jamapsychiatry.2013.27

Durand, G., & Plata, E. M. (2017). The effects of psychopathic traits on fear of pain, anxiety, and stress. Personality and Individual Differences, 119, 198–203. https://doi.org/10.1016/j.paid.2017.07.024

Heck, V., H, C., Driessen, J. M. A., Amato, M., Berg, V. D., N, M., Bhandari, P., Bilbao-Broch, L., Farres-Casals, J., Hendriks, M., Jodzio, A. C., Luque-Ballesteros, L., Schöchl, C., Velasco-Angeles, L. R., Weijer, R. H. A., Rijn, V., M, C., & Jongsma, M. L. A. (2017). Pain Processing in a Social Context and the Link with Psychopathic Personality Traits—An Event-Related Potential Study. Frontiers in Behavioral Neuroscience, 11. https://doi.org/10.3389/fnbeh.2017.00180

Seara-Cardoso, A., Viding, E., Lickley, R. A., & Sebastian, C. L. (2015). Neural responses to others’ pain vary with psychopathic traits in healthy adult males. Cognitive, Affective, & Behavioral Neuroscience, 15(3), 578–588. https://doi.org/10.3758/s13415-015-0346-7

Fedora, O., & Reddon, J. R. (1993). Psychopathic and nonpsychopathic inmates differ from normal controls in tolerance levels of electrical stimulation. Journal of Clinical Psychology, 49(3), 326–331.


METHODS
6) One of the bigger issues is that a series of t-tests were conducted to look for statistical group differences (and without any corrections). Could the authors explain why this approach was chosen, given the chance of obtaining false statistical results and the many problems with frequentist statistics? Wouldn't the use of approaches like MANOVA or Mixed Models be a better choice? It seems strange to discuss small sample size in the limitations section but not actively try to use statistical approaches that may be better in this case. If the findings are robust and reliable, they should remain so across statistical approaches. What would be even more convincing is that the findings remain the same even when the statistical approaches come from different statistical families (e.g., Robust statistics, Bayesian statistics). I request the authors to provide such information to ascertain the reader that the findings are reliable.

Response: Thank you for your suggestion for using a MANOVA test. On consideration of this, we decided that this would not be suitable as we only have one independent variable: psychopathy groups (low and high). Instead, we conducted independent sample’s t-tests using Hedge’s g effect sizes and confidence intervals to correct for our small sample sizes.

7) Sample size calculations are lacking.

Response: Thank you for your comment. We unfortunately did not conduct a sample size calculation prior to the research however we have commented on this in the limitations section of the manuscript and recommended future research do this.

8) The explanation of the empathy task on page 7 is very unclear. The authors should merge it with the task description that comes later to put all the information together.

Response: Thank you for your comment. Both sections have been combined into empathy task description.

DISCUSSION

9) p16: "..we also show that criminal and non-criminal psychopaths share similar physiological characteristics." This claim is misleading, as there were no criminal psychopaths included in the study. Moreover, the idea that psychopathy is a dimensional construct that can be measured in both criminal and non-criminal population is not explained in the manuscript, which is another indication that the theoretical embedding needs to be much more thorough.

Response. Thank you for your comment. The claim comparing criminal and non-criminal psychopaths has been removed, and instead has been used as a discussion point only.

10) Because of the poor embedding in prior research on pain in psychopathy, the discussion seems incomplete and superficial. I also noted that studies in youths are often used to develop arguments in the manuscript, even though there are plenty of studies in adults, who are more similar to the sample studied here. This creates a missed opportunity to position this study more firmly in the literature and highlight its added value.

Response: Thank you for your comment. A more appropriate adult sample study has been used instead.

GENERAL
11) I could not find information about data sharing and access, even though the journal requires this to be stated clearly.

Response: Thank you for your comment. Data will be shared once the reviewer comments have been completed and the manuscript has been resubmitted.

**Reviewer #2:** Thank you for your submission of your article “Psychopathy, pain, and pain empathy: A psychophysiological study" to PLOSE ONE. In the present paper you intended to investigate experienced self-reported as well as psychophysiological pain and empathy for other's pain in participants showing low vs. high psychopathy. I believe that the investigated topic is of value but I have some major concerns about the manuscript. In my opinion, the article does not meet the standards of publication in PLOS ONE in its current state, therefore I recommend a thorough revision before the article is resubmitted to another journal.

First, the article lacks of a clear, systematic and statistical reasonable argumentation in the introduction as well as in the discussion. The line of argumentation is indeed supported with examples from (partly also not so current) literature. However, the described earlier correlations are presented without any statistical indicators. The associations or correlations could be much better interpreted by reporting effect sizes. At times, the argumentation is also unclear and ambiguously explained (e.g., painful facial expression as the ultimate way to show they need help). Furthermore, it is not clear how the results of the present study could explain characteristics of non-criminal and criminal individuals, as this socio-demographic feature was not a part of the study design at all.

Second, and what is particularly concerning, the article is not based on any sound statistical methods. Foremost, the achieved scores of the n = 131 participants, forming the basis of the final sample, were not reported. Therefore, it is not comprehensible how to differentiate the top 20% and lowest values. It could be possible that, with a 4-point Likert scale, as used in this questionnaire, the values were very closely distributed, and the excluded individuals may have hardly differed from those considered as high or low psychopathic participants. Additionally, socio-demographic characteristics would also be of interest here.
The applied method relied solely on t-tests and the assessment of statistical significance. With respect to the new statistics, it is highly recommended to not only evaluate results based on significance (dichotomous, yes or no) but to interpret effect sizes. Moreover, even the chosen alpha level was not specified. Although Cohen ds were reported in later sections (where, given the very small sample size (another point), Hedges' g would be more advisable as this effect size corrects for small samples), the calculation of these standardized mean differences was not described, nor were any thresholds mentioned for interpreting these results (i.e., small/large/medium effects, according to Cohen, 1988). Furthermore, although these Cohen ds were reported in the text and tables, they were not interpreted. In my opinion, this approach is inadequate.

Response: Thank you for your comment. We have added the minimum and maximum values and means and standard deviations of the YPI that participants completed for the low and high psychopathy group to show the difference between the two groups’ scores. In addition, Hedge’s g for sample size has been used to correct for our small sample size.

Finally, the present study was neither preregistered nor is the analysis code or data publicly accessible. Although the authors state that the data would be made available upon request, this approach is also highly concerning with regards to Open Science initiatives.

Response: Thank you for your comment. Data will be shared once the manuscript has been resubmitted.

I hope that these comments were helpful for the future work on the study and I wish the authors good luck with a resubmission!

**Reviewer #3:** The present manuscript investigates the relationship between psychopathy, pain and empathy using both self-reports and psychophysiological measures. The results indicate less self-reported physical pain and empathy in people higher in psychopathy, compared to those lower in psychopathy. Notably, the results obtained using skin conductance response (SCR) to physical pain did not point to differences between people residing in highest and lowest fifth of the psychopathy spectrum.

These main results of the study are highly significant for the discipline as they suggest distinct evaluation of pain in people with psychopathic traits, with only subjective experiences of pain distinguishing people higher and lower in psychopathy. Regarding empathy, both objective and subjective measures suggested distinct responses in people residing in highest and lowest fifth of the psychopathy spectrum.

Both the identified research gaps (see Introduction) and the findings of the study (see Discussion) are properly placed in the context of the previous literature, considering a wide range of studies conducted in the field. Furthermore, the data and analyses fully support the authors’ conclusions. The procedure along with the utilised measures and analyses is described in sufficient detail, ensuring transparency and comprehensibility of the methods used. Finally, the authors discuss the implications of these findings in an intelligible fashion and propose several ideas for future research.

Taken together, the manuscript is well organized, clearly written and scientifically sound. However, there are some points that should be addressed prior to accepting the paper for publication. For details, please see the

attachment.

Taken together, the manuscript is well organized, clearly written and scientifically sound. However, there are some points that should be addressed prior to accepting the paper for publication:

**Abstract:**

- Consider replacing “psychophysiology” with “psychophysiological measures” in the first paragraph.

Response: Thank you for your comment. This change has been made.

- Consider replacing “We suggest psychopathy interventions focus on recognising and empathising with pain.” with “Psychopathy interventions should thus focus both on recognising and empathising with pain.”

Response: Thank you for your comment. This change has been made.

**Introduction:**

- Please add a reference after the first sentence.

Response: Reference added.

- Page 3: Instead of “Why look into pain?”, use the following wording: “There are several
  reasons for investigating pain in the context of psychopathy.” to ensure a smooth transition
  between the first and second paragraph.

Response: Thank you for the comment. This change has been made.

- Page 4: Since *Plos One* is a multidisciplinary journal, I think it would be helpful to shortly
  explain the meaning of “manual and automated pressure algometers” and “Skin Conductance
  Response”.

Response: Thank you for this comment. This change has been made.

- Page 4: The part “reduce the likeliness of socially desirable responding” is somewhat
  confusing and misleading as socially desirable responding represents a bigger problem in self- report measures compared to psychophysiological measures. It should be clear that the part “reduce the likeliness of socially desirable responding” refers to the comparison between mail, web and telephone approaches (see reference Nr. 14), and not to the comparison between self-report and psychophysiological measures.

Response: Amendment has been made, thank you for your comment.

- Page 5: In the sentence “The present study sought to...” please use “self-reported measures” or “self-reports” instead of just “self-reported”.

Response: Thank you. This change has been made.

- Page 5: In my opinion, the two questions (H1 and H2) represent the research questions and not the hypotheses. Thus, I would suggest to rephrase this part as follows: “Following research questions were addressed:
  1) Do people higher in psychopathy experience less intense pain than people lower in psychopathy?;
  2) Do people higher in psychopathy feel less empathy for other people’s pain?
  Alternatively, please rephrase the questions to make them sound more like hypotheses, e.g. If people are higher in psychopathology, then they will experience less intense pain than people lower in psychopathy.

Response: This has been addressed, and hypotheses are now research questions.

**Methods:**

- Page 5: Add “on” before “notice boards”.

Response: Added

- Page 5: The opening parenthesis is missing before YPI.

Response: Added

- Page 5: You write that 131 participants were contacted to take part in the laboratory
  experiment. It would be good to also note how many of them were in the highest and how
  many in the lowest 20% of the psychopathy spectrum.

Response: Figure added

- Page 5: I suggest moving the part “aged between...” right after the parenthesis with the
  gender distribution, namely: “a total of 49 adults (female *n* = 26; male *n* = 23), aged between
  15-55 years old...”.

Response: Change made

- Though both forms are used in standard English, I would suggest using “data were” rather
  than “data was”, since the former is more common in scientific articles.

Response: Thank you for your comment. This change has been made.

- Page 5: Please specify which data (e.g. sociodemographic data) were anonymised. On the
  next page, you write that the data from the tasks were anonymised, but here it remains
  unclear which data were meant.

Response: Demographic, questionnaire, and SCR information added.

- Page 6: It would be helpful to add a figure or a picture to illustrate the setting of the
  experiment. The written explanation is very well written, but a graphical representation would be a nice addition, especially considering the multidisciplinary nature and the wide audience of the journal.

Response: Thank you for your comment. This has been added to supplementary materials.

- Page 6: Please specify which two self-report questionnaires were given to the participants to accommodate a 10-minute stabilisation period for SCR.

Response: Self-report questionnaire names added

- Page 6: To enhance readability, please add “stimulations” after “10 touch pain” and “10 pain threshold”.

Responses: “Stimulations” added

- Page 7: If possible, please indicate *M* and *SD* for the duration of the tasks.

Response: Specific information is unfortunately unavailable as the precise time for each time the task was run for each participant was not recorded.

- Page 7: Please provide a brief explanation for “phalanges” to make the word understandable
  for a wide audience.

Response: Explanation added in brackets

- Page 9: Add a space between 1 and cm2 (in “1cm2 circular probe”).

Response: Space added

- Page 10: Please add the Cronbach’s alpha values for the YPI and its subscales. This has
  been done for other questionnaires, but not for the YPI.

Response: Total Cronbach’s alpha score added for YPI, scores for each subscale are already presented.

- Page 11: When describing the Likert scale used in the IRI, double quotation marks were used, i.e. “does not describe me well” (0) to “describes me very well” (4). In previous questionnaire descriptions, you used single quotation marks. Please revise this for consistency.

Response: Double quotation marks added

- Page 12: In “...and SCR and self-report responses” do you refer to the empathy measure? Or did I misunderstand something?

Response: Thank you for your comment. Responses are computerised numerical rating scale responses from the computerised program, and this has been clarified in the text.

**Results:**

- Italics are not consistently used for letters used as statistical symbol (e.g. page 13, t (47) = 0.51; page 15, paragraph “We also tested if the pain images...”). Please go through the text and make sure italics are consistently used for statistical symbols.

Response: Thank you for your comment. Statistical symbols are now in italics.

- Page 12: The formulation “to bring the skewness to a more normal level” is rather suboptimal. Please consider another wording, e.g. “to ensure that the data follow approximately normal distribution”.

Response: Thank you for your comment. This change has been made.

- Page 13: To better align the wording of the question and the first sentence, consider replacing “greater” with “lower” in the first sentence.

Response: This change has been made.

- Page 14: For consistency, please replace “SC response” with “SCR”.

Response: This change has been made.

- Page 14: In the sentence “High pain intensity was rated...” please add “level” after “at
  significantly higher”, for the sake of clarity.

Response: Thank you for your comment. This change has been made.

- **Discussion:**
- Page 15: In the first sentence “Our study aimed to...” please consider alternative wording for “psychopathy”, e.g. psychopathic traits, psychopathy level.

Response: Wording changed.

- Page 15-16: The sentence “Lastly, we also show...” seems misplaced. In my opinion, it is rather confusing to mention criminal and non-criminal psychopaths this early in the discussion. I suggest deleting the sentence, since it is sufficient to elaborate on this later in the discussion.

Response: Line removed.

- Page 16: In the sentence “Yet, people higher in...” add “is” after “pain threshold which”.

Response: Word added

- Page 16: There is no need to abbreviate callous-unemotional and blood pressure. There are already many abbreviations used on this page and additional abbreviations only interrupt the
  reading flow.

Response: Abbreviations removed

- Page 16: The word “however” appears three times in the last six lines. Please consider
  rephrasing the sentences or replacing “however” with a synonym (e.g. nevertheless) on some
  occasions.

Response: Thank you for your comment. This change has been made.

- Page 18: The internal consistency of some measures was not satisfactory. Please address
  this in the limitations.

Response: This has been added to the limitations, thank you for your comment.

- Page 19: Add a comma after “a finger trapped in a door”.

Response: Comma added

- Page 19: Replace “helps to add to literature” with “adds to literature”.

Response: Wording changed

- Page 19: Instead of “This is important as interventions...” please write “This is of high
  clinical/practical relevance since interventions...”. This way, you avoid repeating the phrase “it is important” and improve the reading flow.

Response: Change made

• Page 19: Consider replacing the sentence “This is important when developing...” with “When developing interventions targeting empathy deficits, a focus should lie on recognising and empathising with pain.”

Response: Change made.

Overall, this is an interesting and well-written study. I hope this feedback will help the authors further improve the quality of the manuscript which, in my opinion, could be accepted after a minor revision

**Reviewer #4:** This experimental study assessed self-reported pain and SCR during (a) self-experienced pressure pain stimulation and (b) while watching pictures of others in pain, comparing results between two groups of students distinguished by their high versus low levels of psychopathic trait. The results show lower reported empathic pain and SCR in the empathy condition, while in the self pain condition the high psychopathic group reported lower high pain but no difference for low pain or for SCR in either pain intensity condition. The topic is interesting. The methods appear to be well done as far as I can see, although some information is missing here (see below).

I have the following main concerns about this study:
1) A main problem is the small sample size – only 25-26 participants in each group.

2) The analysis plan and results presentation is overall rather confusing. This may be due to the authors failing to make 2 important distinctions in their terminology: First, SCR does not measure pain, but physiological activity that may be related to pain. Pain is by definition a subjective experience. Second, the reported pain intensities need to be distinguished from the physical stimulus intensities (pressure) that were used to elicit that pain intensity. As it is, I am unsure throughout the paper what the authors are referring

to.

Response: Thank you for your comment. We have clarified SCR measures and psychophysiological activity in introduction. We have also differentiated reported pain intensities from physical stimulus intensities by referring to reported pain intensities as NRS (numerical rating scale) self-reported responses.

Another problem with the results (but maybe related to the above point) is that all self-experienced pain stimuli were individually adjusted for each participant to low (3/10) or high (6-7/10) intensity. Given this individual adjustment, I do not understand why the authors expected differences in pain ratings between the groups, and how they interpret them. My only idea would be faster habituation in the high psychopathic group, but this topic is not discussed anywhere. Would not it make more sense to compare the physical pressure intensities in this setup? Also it would be helpful to present the results visually (one could skip the SCR timing images if necessary).

Response: Thank you for your comment. A line about pain habituation has been added in the discussion.

3) There is already quite some literature in relation to psychopathy on the topics of SCR, pain and empathy. The presentation of this literature appears to be insufficient. For example, I am missing the article by Meffert et al. (2015) showing that pychopathic individuals can show relatively normal empathy when explicitly asked to feel with the other person. The authors need to explain more convincingly what is new about their study, and also explain more clearly what their hypotheses are and why.

Response: Thank you for your comment. We have added paragraph with research looking at empathy in psychopathy, including mentioned references.

4) Some information is missing on methods: E.g. what electrodes and what gel was used to measure SCR? How was the SCR signal standardized to be comparable between participants (Boucsein et al. 2012)?

Response: Thank you for your comment. Information has been added regarding gel in electrodes. We had added more information regarding the SCR signal procedure in the methods section.

5) The manuscript seems to contain many typos and there are some problems with the English language throughout.

Response: Thank you for your comment. Extra attention has been paid to this.

Minor points:

Abstract:
The abstract should contain more methods details, e.g. names of the questionnaires, used pain methods.

Response: Thank you for you comment. The names of the questionnaires along with the type of pain used has been added to the abstract.

One of the last sentences (lack of awareness to ones’ body sensation may underlie emotional impairments in psychopathy) seems like a new hypothesis, not a conclusion that can be drawn from the results of this study.

Response: Thank you for your comment. We have altered this so that it is obvious that our results are adding to the existing literature on the topic.

Introduction:
p. 3: The sentence “This raises the question of whether there is a link between psychopathy, empathy for others, and experiencing pain“ seems to give a wrong impression – there is already quite some research on the topic of psychopthy, pain perception and empathy.

Response: Wording changed

p. 4: Why do self-report measures reduce the likeliness of socially desirable responding?

Response: Statement has been amended

The hypotheses mentioned at the end of the introduction do not follow logically from the introduction and are confusing because it is not clarified whether they refer to self-report or SCR results. Given the introduction one would rather expect a hypothesis on the relation between self-report and SCR results.

Response: Research questions have been changed to include self-report measures and SCR

Methods:
p. 5: Sample: The authors screened N=369 students for psychopathy. How is it possible that as many as N=131 students fulfilled the criterion of upper/lower 20% of psychopathy spectrum?

Response: This is possible as many participants fell within the top and bottom 20% of the YPI by having the same or similar scores on the measure thus residing in the lower and upper range of the spectrum, e.g. multiple people scoring the most and least possible on the measure.

p. 6: Pain test: a VAS does not have numbers/ticks in between, but a SAM does. Please clarify if you used a SAM / NRS (numerical rating scale) or a VAS.

Response: VAS changed to NRS for consistency. A SAM was also used as a visual representation of the NRS and has remained in the text.

p. 10: Empathy task: Why was a different pain scale used compared to the pain task (different range 0-100, different end point descriptors)?

Response: Whilst the scales are related (being decimal and divisible by factor of 10) we used the 0-100 scale for the pain task because we anticipated a need for a greater resolution to capture nuanced differences in pain experience between trials. After reviewing the experimental program, both tasks used 0-100 scoring scale, and this has been amended.

Please clarify and credit who created the pictures. Where they created by Fallon et al. (2015)?

Response: Thank you for your comment. The images were first used in Fallon, N., Li, X., & Stancak, A. (2015). Pain Catastrophising Affects Cortical Responses to Viewing Pain in Others. PloS one, 10(7), e0133504-e0133504 which had now been clarified in the manuscript.

Results:
p. 13: Why are average pain results >10 when pain intensity was assessed on a 0-10 scale (see methods)?

Response: Thank you for you comment. I think this might be an error on our part. The methods have been changed to reflect the scoring method (0-100).

p. 14: Manipulation checks: A non-significant trend is presented as if it were a sign result.

Discussion:
The conclusion that the high psychopathy group reported lower self-experienced pain is true only for the high pain condition, and the meaning of this result remains unclear. It should be clarified (already in the methods) whether the low pain condition is actually a no pain condition.

Response: Thank you for your comment. We have corrected the wording and the ‘no pain’ condition is also referred to as a touch condition as participants were given a touch sensation, which generated zero pain, rather than it being a low pain condition.

p. 16: “Yet, people higher in psychopathy did not choose significantly higher levels of pain when selecting their pain thresholds…” the phrase suggests that the authors failed to distinguish pain intensity (a subjective rating) from physical stimulus pressure intensity (an objective rating).

Response: Thank you for your comment. This sentence has been removed since it may have been over-interpreted on our behalf.

References:

Boucsein W, Fowles DC, Grimnes S, Ben-Shakhar G, roth WT, Dawson ME, Filion DL; Society for Psychophysiological Research Ad Hoc Committee on Electrodermal Measures. Publication recommendations for electrodermal measurements. Psychophysiology. 2012 Aug;49(8):1017-34. doi: 10.1111/j.1469-8986.2012.01384.x. Epub 2012 Jun 8. PMID: 22680988.

Harma Meffert, Valeria Gazzola, Johan A. den Boer, Arnold A. J. Bartels, Christian Keysers, Reduced spontaneous but relatively normal deliberate vicarious representations in psychopathy, Brain, Volume 136, Issue 8, August 2013, Pages 2550–2562, <https://doi.org/10.1093/brain/awt190>

**Reviewer #5:** This paper examines whether people scoring high and low on the psychopathy scale differ in their SCR to pain stimulus, their self-reports on pain, and whether high and low people score differently when it comes to empathizing with other’s pain.

This research has major flaws regarding study design, empirical strategy, and statistical analysis. I only list the major issues here:

1. Just because someone who scores high on the psychopathy scale reports low self-reported pain, we cannot conclude that these people have dissociation between their bodily sensations and what they experience. This self-report is confounded with their high psychopathy level, which can also impact their image concerns and their manipulative motives. E.g., even though they experience high pain, they will not report it to be seen as tough.

Response: Thank you for your comment. We have discussed this as a drawback to our research in the limitation section.

2. The sample size is very small for all these pairwise analyses. There is no sample size calculation presented.

Response: Thank you for your comment. Hedge’s g correction has been used for effect size and confidence intervals. We commented on the lack of a sample size calculation in the limitations section of our manuscript and recommended future research conduct a sample size calculation.

3. There are at least ten pairwise comparisons in the paper and no adjustment for them. Bonferroni correction would need to adjust alfa – level to 0.05/10 which would eliminate most of the results.

Response: Thank you for your comment. We have used Hedge’s g correction to help account for the small sample size as we only have one DV and only 2 groups so cannot conduct an ANOVA to use Bonferroni correction. There were no significant changes when using Hedge’s g correction.

4. Weird that the reported degrees of freedom for the independent sample t-tests are sometimes 47 (which is okay, given the 26 and 23 participants in the two groups) and sometimes 45 (which is strange).

Response: Reported degrees of freedom of 45 are due to only having 25 low and 22 high participants’ data for this particular test. This has been explained in the data analysis section of the manuscript.

5. I could not reproduce the t-test statistics based on the reported means and SDs.

Response: Statistical tests have been re-ran and are in alignment with the numbers provided in the previous manuscript and have been updated in the revised manuscript with Hedge’s g correction for our small sample size.

6. Strange that in some cases SE and not SDs for means are reported and yet independent t-statistics are presented.

Response: Thank you for your response. Standard deviations are now presented along with t-tests.

7. Based on the reported means, SDs and Ns, I could not reproduce the reported p-values.

Response: Statistical tests have been re-ran and are in alignment with the numbers provided in the previous manuscript. Statistical tests have been updated in the revised manuscript with Hedge’s g correction for our small sample size.
