# Supplementary material for: Psychopathy, pain, and pain empathy: A psychophysiological study
Source: PLoS One. 2024 Jul 5;19(7):e0306461. doi: 10.1371/journal.pone.0306461 (PMC11226074; doi:10.1371/journal.pone.0306461)
Supplement: Attachment — Submitted filename: Response to reviewers FINAL.docx [file pone.0306461.s007.docx]

Dear reviewers,

Thank you very much for the comments you have made on our manuscript. We appreciate the feedback and have hopefully addressed all the comments that have been made to the correct standard. Below, you will find each of the comments that we received and the action we have taken.

Thank you for your time and effort when reviewing our work.

Reviewer #2: The authors improved their manuscript and addressed the reviewer's comments appropriately. Please italicize statistical expressions in the methods section.

Response: Thank you for your comment and taking the time to review our manuscript.

Reviewer #3: Abstract:
• When introducing the abbreviation YPI, write out the full term first and then write the abbreviations in the parenthesis.

Response: Thank you for your comment. This change has been made.

Introduction:
• Line 53: Replace “stem” with “stems” as it refers to inability (i.e. inability stems).

Response: Thank you. Change has been made.

• Line 62-63: The part “however, when asked to empathise…” is unclear. I think this is because a subject is missing. Please revise.

Response: Thank you for your comment. We have revised this sentence.

• Line 86: The sentence should be rephrased – measures cannot correlate. Did you mean: “Psychopathy was correlated with increased pain tolerance in studies using self-report measures.”? Please check.

Response: Thank you for your comment. This change has been made.

• Line 120: I suggest adding a reference for “dual harm model”.

Response: Thank you for your comment. This reference has been added.

Methods:
• Line 237: The opening quantitation mark is missing in the example item for impulsiveness.

Response: This has been added.

• Line 242: This indention is bigger that others.

Response: This has been amended.

• Line 268-269: Please use italics for subsample size (n).

Response: Italics have been added.

• Line 278: I think “high” should be replaces with “higher”.

Response: This change has been made.

Results:
• Line 287: I suggest putting (Z = 1.94) after “directly experienced pain”.

Response: This change has been made.

• Line 301: “Ehen given higher levels of pain” should be deleted as this part already appears at the beginning of the sentence.

Response: This has been removed.

• Line 348: “images” should be deleted after “pain”.

Response: This has been removed.

Discussion:
• Line 362: Replace “its” with “their” since you are referring to traits.

Response: This has been changed.

• Line 386: To improve readability, add “that” after “suggest”.

Response: This has been added.

• Line 388-389: I find “other possible psychological areas” too general. Please choose a more specific term (e.g. other psychological areas related to pain and empathy).

Response: This has been added.

• Line 457: Replace “were” with “was” since you are referring to internal consistency.

Response: This has been changed.

Reviewer #4: The literature review in the introduction is much improved and develops the research question more convincingly now. However, several of my other previous major comments have not been adressed sufficiently by the authors, including:

1) Wrong interpretation of the pain rating results: The authors compare self-pain ratings (NRS) between the groups for pressure stimuli whose intensity was individually adjusted so that they induced the same pain rating in all participants in the first part of the experiment. The finding that these high pain stimuli later induced lower pain ratings in high versus low psychopathic individuals (p = .03) does not indicate overall lower pain perception in high psychopathic individuals, but a reduction in pain perception across the experiment. This is an interesting result, possibly due to faster habituation or, alternatively, different reporting as high psychpathic individuals may choose not to acknowledge their pain as the experiment proceeds.

Response: Thank you for your comment. Pain habituation has been addressed in the discussion, and the limitation of people with psychopathy not admitting to pain due to masking or wanting to appear tough.

However, to answer the first main research question of the study – whether high psychopathic individuals experience less intense pain compared to low psychopathic individuals – the objective volts leading to similar perception of high pain during pain calibration are at least equally if not more relevant, given that participants are less likely to manipulate their answers during a random staircase stimulation session. These results are reported in Line 481-483 and show a non-significant trend (p = 0.05) in the right direction (lower pain sensitivity in high psychopathic individuals during pain calibration), but this is not a manipulation check but a major result, and it needs to be correctly presented and discussed.

Response: Thank you for your comment. Since running the ANOVA, this result is no longer in the right direction (towards significance) and so have not been regarded as a major result as previously suggested.

2) In consideration of the small sample size, the authors should have replaced multiple t-tests by a single ANOVA, and should additionally have applied alpha-corrections to the post-hoc contracts following that ANOVA to render their findings more reliable, but this was not done in spite of reviewer comments.

Response: Thank you for your comment. We have ran the ANOVA to make findings more reliable, and altered the discussion based on those changes.

3) Related to the above points, I do not agree with the main conclusion of the study: Even if the weak significant group difference regarding self-pain ratings found in the study would survive the more conservative approach of ANOVA + alpha correction, overall the main message from the data seems to be that group differences are evident in empathy much more than in self-perceived pain. This also speaks against the claim that lack of awareness of body sensations is the cause of low empathy in psychopaths.

Response: Thank you for your comment. We conducted the recommended ANOVA and self-pain ratings did not survive this test, thus, this is no longer a conclusion of the study.

4) Wrong terminology is still used in many places, e.g.
* No correct distinction between nociceptive stimuli (defined by objective volts) and pain (a subjective response to those stimuli) is creating confusion throughout the manuscript,

Response: Thank you for your comment. Adjustments have been made when referring to nociceptive stimuli and subjective responses to pain.

e.g.
Line 586: „Yet, people higher in psychopathy did not choose significantly higher levels of pain when selecting their pain thresholds.“ should be rephrased to „...did not choose significantly higher levels of pressure when selecting their pain threshold“.

Response: Thank you for your comment. This has been addressed.

* SCR is still described as a measure of pain when in fact it is a measure of arousal, which can also be caused by stimuli of positive valence – a relevant point when showing pictures of others in pain to psychopaths. This problem is already evident in the way the research questions are phrased at the end of the introduction.

Response: Thank you for your comment. This change has been made.

* The authors mention pain tolerance in several places, e.g. Line 639: „...we predicted that individuals with psychopathy would have… higher pain tolerance themselves“. Pain tolerance (i.e. the intensity of pressure and/or amount of time a painful stimulus is tolerated before aborting it) was not measured in this study. Only pain perception was measured.

Response: Thank you for your comment. We have changed ‘pain tolerance’ to ‘pain perception/physical pain/greater levels of pain’.
