# Supplementary material for: Psychopathy, pain, and pain empathy: A psychophysiological study
Source: PLoS One. 2024 Jul 5;19(7):e0306461. doi: 10.1371/journal.pone.0306461 (PMC11226074; doi:10.1371/journal.pone.0306461)
Supplement: Attachment — Submitted filename: Response to reviewers FINAL.docx [file pone.0306461.s008.docx]

Dear reviewers,

Thank you for taking the time to re-review our manuscript. We greatly appreciate you taking the time to provide feedback. We have acknowledged the mistakes we made in the previously submitted manuscript and have hopefully now addressed all the comments that have been made to the correct standard. Below, you will find each of the comments that we received and the action we have taken.

Thank you again for reviewing our work.

Reviewer #3: It is clear that the authors made a considerable effort to incorporate reviewers‘ feedback. In my opinion, there are now only some small issues (mostly grammar mistakes) in the manuscript that still need to be addressed:

Lines 62-63: Please ensure tense consistency (either: were asked – they showed, or: are asked – they show).

Response: Thank you for your comment. These changes have been made.

Line 287: If I understand the sentence correctly, “significantly high” should be replaced by “significantly higher”.

Response: Thank you for your comment. This change has been made.

Line 332: Did you mean “as less painful”? Please check.

Response: Thank you for picking up this mistake – the change has been made.

Lines 337-339: I’m having difficulties understanding the sentence “Post hoc t-tests…”. Please check and rephrase for clarity.

Response: Thank you for your comment. This change has been made.

Line 386: Replace “despite our findings” with “contrary to our findings” or “in contrast to our findings”.

Response: Thank you for your comment, this change has been made.

Line 391: I think “either” is not needed here.

Response: Thank you, this has been removed.

Lines 397-399: The part “or a masking or psychological feature as psychopathy boasts deceptive and manipulative capabilities” is unclear to me. Please revise.

Response: Thank you, this has been revised.

Lines 401-402: Consider rephrasing the sentence as follows “Future research should examine this link more closely.” Also, in the preceding sentence, replace “ones’” with “one’s”, and “in psychopathy” with “in people with high psychopathic traits”.

Response: Thank you, these changes have been made.

Lines 412-413: Consider omitting “entirely”.

Response: This change has been made.

Lines 413-414: Consider rephrasing the sentence as follows: ”Although our finding was non-significant, the link between alexithymia and psychopathic traits should be explored further, as previous research found a strong support for this link (e.g. 4)”.

Response: Thank you, this change has been made.

Lines 444-445: Please check the part “a accept higher levels…” and revise as appropriate.

Response: Thank you, this has been revised.

Line 471: Consider rephasing as follows: “Lastly, although appropriate statistical tests were used…”.

Response: Thank you, this change has been made.

Reviewer #4: I am sorry to have to say that most of my previous major concerns have not been met in this R2 version of the manuscript:

1) Wrong interpretation of the pain rating results: The authors compare self-pain ratings (NRS) between the groups for pressure stimuli whose intensity was individually adjusted so that they induced the same pain intensity rating in all participants in the first part of the experiment. Given this methodological approach, the objective pressure (measured in volts), rather than the subjective pain ratings (measured with a NRS) should be the main outcome of interest for the self-pain condition. It is therefore positive that the objective pressure results are now featured as a result rather than a manipulation check in this revised version of the manuscript.

Response: Thank you for your comment.

However, the revision is not thorough and comprehensible enough. For example, the individually adjusted stimulus intensities are an important feature of the study and should be mentioned already in the abstract, otherwise the results cannot be understood correctly.

Response: Thank you for your comments. The fact that pain is individually adjusted for each participant has been included in the abstract.

Also, the logical reasoning of the manuscript throughout Methods, Results and Discussion is still that the pain ratings (NRS) rather than the pressure intensities (volts) should show a significant effect. Overall it appears that the authors do not understand the logic of their own experimental design.

Response: Thank you for your observation. I think as a team, internally, we were used to using the word ‘pain’ to encompass both the pressure stimuli and the subjective pain ratings while conducting the experiment, and then during the writing process, we still used them interchangeably rather than separating the two distinct subjects. Thank you for pointing out the distinction, and this has now been rectified throughout the manuscript.

2) Wrong use of terminology: The authors still fail to correctly distinguish between pain, which is by definition a subjective experience, and the objective stimulus pressure administered to induce that pain. This problem continues to cause confusion throughout the manuscript, as shown in the following example:

Line 280: „Additionally, we tested whether people higher in psychopathy accepted greater levels of high-pressure nociceptive pain (measured in volts) compared to the low psychopathic group…with… pain intensity level (high pain) as the dependent variable“.

This sentence is confusing and wrong because a) volts are not a unit of pain but of pressure, b) indeed, the correct dependent variable here is pressure, not pain, and c) the phrasing „accepted“ insinuates that pain tolerance was measured when in fact participants simply had to rate how intense their pain perception was with each stimulus. This misleading and confusing wording is used throughout the manuscript, e.g. in the results section (Line 326-329). An example for a more correct phrasing would be e.g. „To test whether people higher in psychopathy required objectively more intense pressure stimuli (measured in volts) to report the same subjective pain intensity as the low psychopathic group, we performed a 2-way mixed ANOVA…“.

Response: Thank you for your comment. After reading your example, we understand where we went wrong. We have amended this throughout the manuscript.

3) A new concern in this R2 version is that the presentation and discussion of results regarding self-pain ratings is contradictory throughout the manuscript:
The abstract and the response to reviewers letter suggest that the revised data analysis using ANOVA instead of multiple t-tests did not find a significant between-group difference in self-pain ratings any more:

Line 33 Abstract: „People higher in psychopathy did not self-report feeling less physical nociceptive pain compared to people lower in psychopathy“.

Response: Thank you for this observation, this mistake has been altered.

Reponse to reviewers: „We conducted the recommended ANOVA and self-pain ratings did not survive this test, thus, this is no longer a conclusion of the study“.

In contrast, the results session does report a highly significant between-group difference in self-pain ratings:
Line 316: „...the high psychopathy group reported less pain (M = 33.96, SD = 1.70) compared to the low psychopathy group (M = 40.40, SE = 1.60), t (47) = 2.75, p <.001“.
The high significance of this effect also contradicts the R1 version of this manuscript, where the t-test obtained only p=.03 (Line 429 in R1 manuscript).

Response: Thank you for your comment. Upon inspection, we made an error and an extra 0 was added to <.001 and instead it should be <.01, which has now been adjusted.

This significant result is referred to throughout the Discussion, e.g.
Line 480 Discussion: "Additionally, we found people in the high psychopathy group self-reported experiencing less intense physical pain compared to people in the low psychopathy group".
But then again no effect seems to have been found:
Line 549: „we predicted that individuals with psychopathy would have reduced empathy for others’ pain as well as a accept higher levels of pain themselves. Our findings show support for the former, but not the latter„.

Response: Thank you for this observation, this has now been altered to show support for both predictions based on our significant finding.

While in the conclusion the significant effect reemerges:
Line 593 Conclusion: „...psychopathic traits were associated with a reduced perception of nociceptive pain“.

This presentation is completely contradictory and inacceptable.

Response: Thank you for being so observant, and we apologise for the confusion on our behalf and the error we made. We did in fact find a significant result and this has been consistently altered throughout the manuscript. We again apologise for our mistakes and thank you for taking the time to point out our errors.
