# Supplementary material for: Psychopathy, pain, and pain empathy: A psychophysiological study
Source: PLoS One. 2024 Jul 5;19(7):e0306461. doi: 10.1371/journal.pone.0306461 (PMC11226074; doi:10.1371/journal.pone.0306461)
Supplement: Attachment — Submitted filename: Reviewer comments FINAL.docx [file pone.0306461.s009.docx]

Dear Reviewer #3,

Thank you for again taking the time to review our document. We have made the changes you have recommended, and you will find each of the comments that we received and the action we have taken.

Thank you again for reviewing our work.

Reviewer #3: The authors have adequately addressed the issues found in the previous version. Overall, the revised manuscript reads well and is much clearer. I have identified only a small number of minor issues. I suggest authors resolving these issues to further improve the readability and clarity of the manuscript.

Abstract
Remove „growing“ in the penultimate sentence. Alternatively, you could write „We also show support for the theory of dual harm, which has been receiving increasing attention.“

Response: Thank you, this has been amended.

In the last sentence, write either „consequently“ or „thus“. Using both is redundant.

Response: Thank you, this has been removed.

Introduction
Page 4, line 85: Add a verb, e.g. “Fourth, mixed findings can be found in the current literature on psychopathy…” or “Fourth, current literature suggests mixed findings…”.

Response: Thank you, this change has been made.

Page 5, line 124: Consider rephrasing as follows “…with little research looking into (or investigating) psychological aspects.” to avoid word repetition.

Response: Thank you, this change has been made.

Discussion
Page 22, line 513-515: Consider rephrasing as follows “…suggest conducting an a priori power analysis and testing psychopathy groups within larger samples to increase statistical power.”

Response; Thank you, this change has been made.

Page 22, line 527: For better clarity, replace “these traits” with “psychopathological traits”.

Response: Thank you, this change has been made.

Page 22, line 532ff: Rephrase as follows “In conclusion, our study provides support for the dual harm model by demonstrating a diminished perception of pain in people higher in psychopathy. Additionally, we contribute to the current literature by showing that high psychopathic traits relate to problems in empathising with others’ pain.”

Response: Thank you, this change has been made.
